# Evaluation of Clinical Outcomes and Graft Patency Following Venous Resection and Vascular Reconstruction Using a Recanalized Ligamentum Teres Hepatis Graft in Pancreaticoduodenectomy

**DOI:** 10.1245/s10434-026-19379-6

**Published:** 2026-03-03

**Authors:** Wen-Tao Zhu, Zhi-Wei Liu, Qiang Wei, Fan Zhang, Hai-Bin Ji, Jian-Yong Cui, Qiang-Pu Chen

**Affiliations:** 1 Department of Hepatobiliary Surgery, Binzhou Medical University Hospital, Binzhou, China; 2 Department of Clinical Nutrition, Binzhou Medical University Hospital, Binzhou, China

**Keywords:** Ligamentum teres hepatis, Vascular graft, Portal vein, Superior mesenteric vein, Pancreaticoduodenectomy

## Abstract

**Purpose:**

Our objective was to evaluate clinical outcomes and vascular graft patency following portal vein/superior mesenteric vein (PV/SMV) reconstruction using recanalized autologous ligamentum teres hepatis (LTH) grafts.

**Methods:**

This study enrolled 387 patients, stratified into three groups: (1) pancreaticoduodenectomy alone, (2) pancreaticoduodenectomy with vascular resection and reconstruction via end-to-end anastomoses or lateral venorrhaphy, and (3) pancreaticoduodenectomy with PV/SMV resection and reconstruction using autologous LTH grafts. This retrospective study compared operative time, intraoperative blood loss, postoperative complication rates, mortality, and length of postoperative hospital stay across these groups.

**Results:**

The study included 336 patients who underwent pancreaticoduodenectomy, 23 who underwent pancreaticoduodenectomy with vascular resection and reconstruction via end-to-end anastomosis or lateral venorrhaphy, and 28 who underwent pancreaticoduodenectomy with PV/SMV resection and reconstruction using autologous LTH grafts. The group using autologous LTH grafts exhibited an operative time of 484.86 ± 103.77 (285–685) min; intraoperative blood loss of 236.79 ± 141.95 (80–800) mL; a postoperative complication rate of 42.86%; 30-day mortality rate of 7.14%; and postoperative hospital stay of 20.82 ± 8.25 (9–49) days. Statistical analysis revealed a significantly longer operative time in the autologous LTH grafts group than in the other groups (*p* < 0.001), with no significant intergroup differences in blood loss, postoperative complication rates, mortality, or hospitalization. Partial thrombosis involving ≤50% of vessel diameter without obstruction was observed in four cases involving autologous LTH grafts. All reconstructed vessels maintained 100% patency throughout the follow-up period.

**Conclusions:**

Using autologous LTH grafts for PV/SMV reconstruction during pancreaticoduodenectomy was safe and feasible, demonstrating favorable vascular graft patency rates and supporting its role as a viable alternative conduit for vascular restoration.

**Supplementary Information:**

The online version contains supplementary material available at 10.1245/s10434-026-19379-6.

Cancers of the lower bile duct, pancreatic head, and ampulla of Vater frequently infiltrate the portal vein (PV) and/or superior mesenteric vein (SMV). Pancreaticoduodenectomy (PD) with concurrent PV and/or SMV resection has become a well-established, safe, and effective standard surgical approach.^1–5^ Three reconstruction techniques are commonly employed for PV and SMV: end-to-end anastomosis, lateral venorrhaphy, and the use of vascular grafts.^3,6–9^ Current vascular replacement options comprise synthetic prostheses, autologous grafts, and allogeneic homografts, each associated with distinct clinical limitations. Consequently, the development of novel vascular reconstruction materials for PV and SMV repair has become a critical necessity in modern hepatopancreatobiliary surgery.

The ligamentum teres hepatis (LTH), a remnant of the obliterated left umbilical vein from the embryonic period, can undergo dilation and recanalization following surgical harvest. Since 2003, LTH has been effectively utilized at our institution as an autologous venous graft for reconstructing major abdominal veins, including the PV, SMV, inferior vena cava (IVC), splenic vein, and hepatic veins (HV).^10,11^ To date, no studies have systematically analyzed the postoperative complication rates and graft patency of LTH used for PV or SMV reconstruction in patients undergoing PD with vascular resection. This study aimed to evaluate the application of LTH in PD with PV or SMV resection and reconstruction by assessing clinical outcomes and vascular graft patency.

## Methods

### Patients and Grouping

The experimental protocol was approved by the ethics committee of Binzhou Medical University Hospital (approval no. 2019-LW-023); written informed consent was obtained from all participants. Patients who underwent open PD between January 2003 and June 2024 at the Binzhou Medical University Hospital were retrospectively selected. Patients were categorized in three groups: (i) those who underwent PD without PV or SMV resection (PD group), (ii) those who underwent PD with PV or SMV resection and reconstruction via end-to-end anastomosis or lateral venorrhaphy (PD-VR group), and (iii) those who underwent PD combined with PV or SMV resection and reconstruction using the autologous LTH graft (PD-VRR group). A retrospective review of medical and radiological records was conducted for all included patients. Data on patient demographics, clinical characteristics, perioperative variables, and postoperative graft patency were systematically collected.

The inclusion criteria were as follows: (i) patients who underwent PD for neoplasms involving the biliary tract, pancreatic parenchyma, ampullary region, or duodenum and (ii) patients with complete clinical data.

The exclusion criteria were (i) PD combined with resection of other organs, (ii) laparoscopic PD, (iii) total pancreatectomy, and (iv) incomplete case data.

### Surgical Procedures

An upper midline transumbilical abdominal incision was made under general anesthesia with endotracheal intubation. PD resection included 30–50% distal gastrectomy, cholecystectomy, mid-to-distal common bile duct resection, excision of the pancreatic head and duodenum, and proximal jejunal resection measuring 15–20 cm. Lymphadenectomy of the pancreatic head and the hepatoduodenal ligament was also performed. Digestive tract continuity was restored using Child reconstruction, which included end-to-side pancreatojejunostomy, end-to-side hepaticojejunostomy, antecolic gastrojejunostomy, and Braun enteroenterostomy positioned 12–15 cm distal to the gastrojejunal anastomoses in selected cases. A jejunal feeding tube was placed into the efferent limb through either a jejunostomy or nasojejunal intubation. Pylorus-preserving PD was performed in selected patients. Total operative duration, PV clamping time, and intraoperative blood loss were recorded.

#### PD-VR Group

**Fig. 1:**
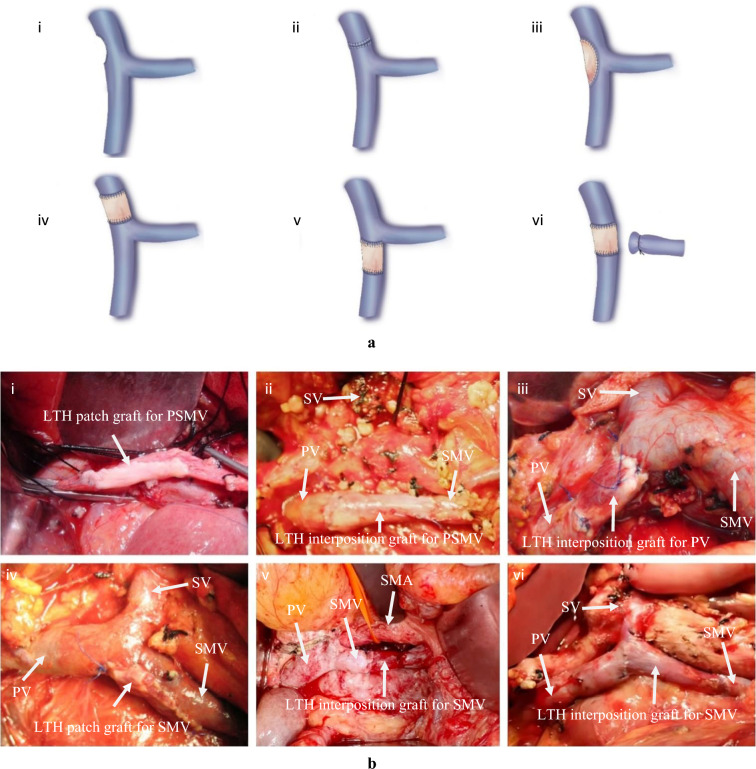
Schematic design and surgical implementation of surgery. **a** Schematic diagram of the surgical approach: (ⅰ) portal vein (PV)/superior mesenteric vein (SMV) reconstruction via lateral venorrhaphy; (ⅱ) PV/SMV reconstruction via primary end-to-end anastomosis; (ⅲ) PV/SMV reconstruction with ligamentum teres hepatis (LTH) patch graft; (ⅳ) PV reconstruction with LTH interposition graft; (ⅴ) SMV reconstruction with LTH interposition graft; (ⅵ) portal-superior mesenteric vein (PSMV) reconstruction with LTH interposition graft. **b** Intraoperative photograph: (ⅰ) Using LTH as a patch graft for PSMV reconstruction in a 70-year-old male patient with pancreatic adenocarcinoma; (ⅱ) using LTH as a interposition graft for PSMV reconstruction in a 62-year-old male patient with ampullary carcinoma; (ⅲ) using LTH as a interposition graft for PV reconstruction in a 47-year-old female patient with cholangiocarcinoma; (ⅳ) using LTH as a patch graft for SMV reconstruction in a 56-year-old male patient with pancreatic adenocarcinoma; (ⅴ) using LTH as a interposition graft for SMV reconstruction in a 59-year-old female patient with pancreatic adenocarcinoma; (ⅵ) using LTH as a interposition graft for SMV reconstruction in a 71-year-old female patient with pancreatic adenocarcinoma

Dissection was carried out as mentioned previously. The PV, SMV, and splenic vein were mobilized and clamped as necessary. The surgical specimen was resected en bloc along with the involved venous segment(s) of PV/SMV. For lateral resection of the venous wall involving less than one-third of the vascular circumference, repair was performed using continuous sutures. In cases requiring segmental venous resection with a defect length of <3 cm, vascular continuity was restored by in situ end-to-end anastomoses. All vascular anastomoses were performed using 5–0 polypropylene sutures in a continuous running technique. Figure 1a presents a schematic illustration of vascular reconstruction following PV/SMV resection.

#### PD-VRR Group

**Fig. 2:**
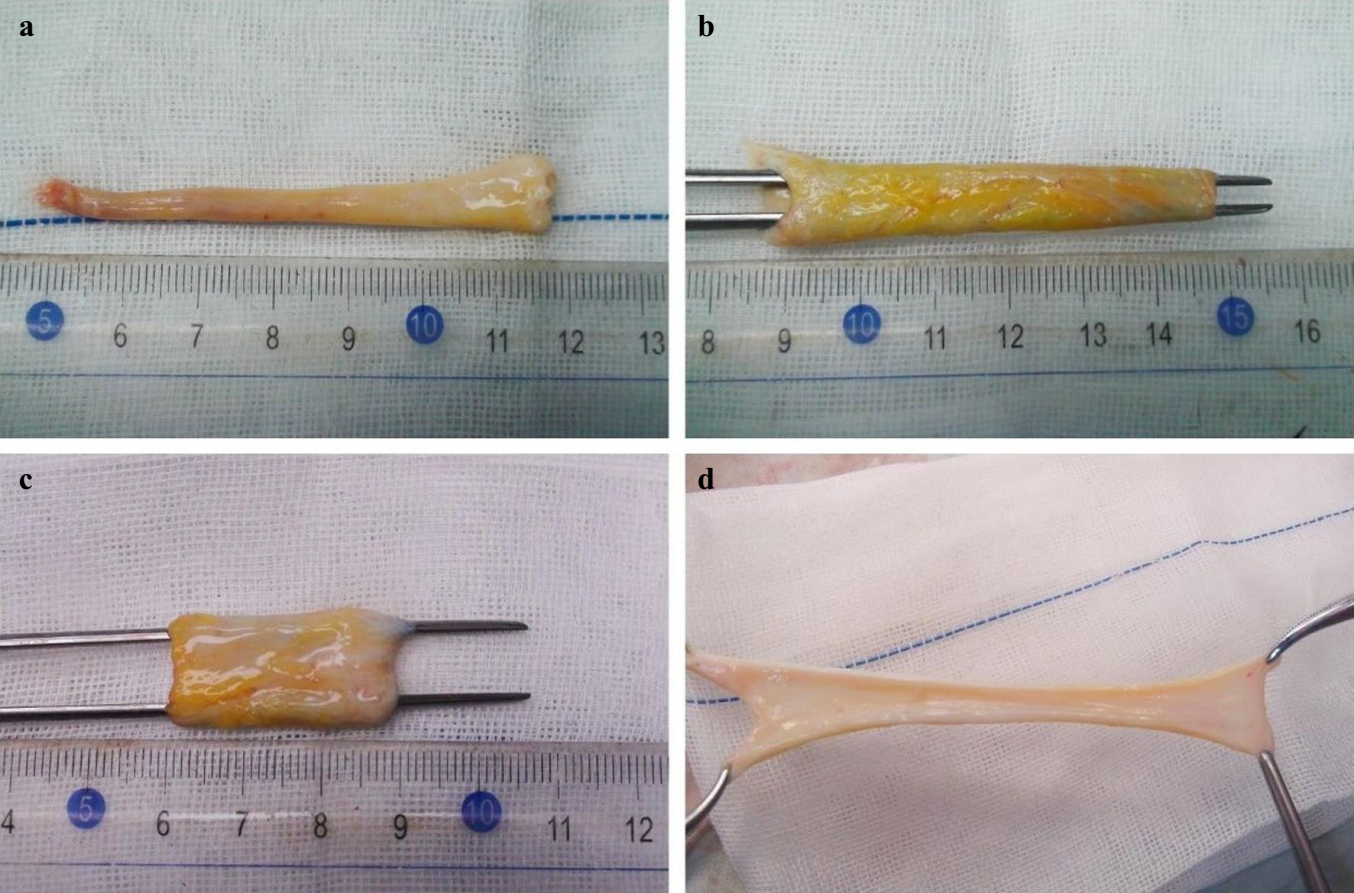
Dilated and trimmed procedure of ligamentum teres hepatis (LTH). **a** Resected LTH, **b** dilated LTH, **c** LTH interposition graft, and **d** LTH patch graft

The dissection was performed as mentioned previously. The necessity for PV/SMV resection followed by reconstruction using an autologous recanalized LTH graft was evaluated and confirmed. The LTH was initially mobilized and harvested, after which adherent adipose tissue was meticulously debrided to expose the graft (Fig. 2a). The residual cavity of the LTH was identified, and the lumen was expanded (Fig. 2b). The detailed methodology of the dilation technique has been described previously.^12^ Following recanalization, the LTH was either trimmed into a tubular graft or incised longitudinally to create a sheet (Fig. 2c, d). The trimmed LTH was subsequently preserved in heparinized saline until further use (Video 1). The procedure for PV/SMV resection followed the same protocol used in the PD-VR group.

Vascular reconstruction was performed immediately after removal of the specimen. When the excised portion of the PV/SMV exceeded one-third but was <50% of the venous circumference, the LTH was utilized as a lateral patch graft to reconstruct the vascular wall defect (Fig. 1b ⅰ, ⅱ). If the excised PV/SMV segment exceeded 3 cm in length, the LTH was employed as an interposition graft to reconstruct the vascular defect (Fig. 1b ⅲ–ⅵ). All vascular anastomoses were constructed using continuous 5– polypropylene sutures. Prior to completing the anastomoses, the recipient venous stumps and graft conduit were irrigated with heparinized saline. The PV clamp was then released, followed by irrigation with heparinized saline to clear potential thrombi and ensure luminal patency (Video 2). On completion, the anastomoses were checked for leaks, and the graft vessel was assessed to confirm pulsatile graft perfusion and the absence of torsional deformity or axial tension.

### Postoperative Treatment

All patients were administered postoperative parenteral nutrition (PN) support for 3–5 days following surgery. Early enteral nutrition was commenced on postoperative day (POD) 1 through a jejunostomy feeding tube, with a daily incremental increase in calorie intake. A gradual transition from PN to complete enteral nutrition was achieved through progressive supplementation with enteral feeds until complete PN discontinuation. In the PD-VR and PD-VRR groups, patients without clinically significant bleeding risk received standard anticoagulation therapy with subcutaneous low molecular weight heparin (4100 IU every 12 h) from POD 1–7. Aspirin or low-dose warfarin therapy was initiated on POD 8 for all patients. This therapeutic protocol was continued for 3 months postoperatively.

### Assessment of Clinical Outcomes

Intraoperative parameters, including operative time, PV clamping time, and blood loss, were recorded and analyzed. Postoperative complications were evaluated using the standard guidelines of the International Study Group of Pancreatic Surgery and the complication classification by Tan et al.^13^ and Dindo et al.^14^ Postoperative complications, 30-day mortality, and length of postoperative hospital stay were compared across the three groups.

### Follow-Up and Vascular Patency

**Fig. 3:**
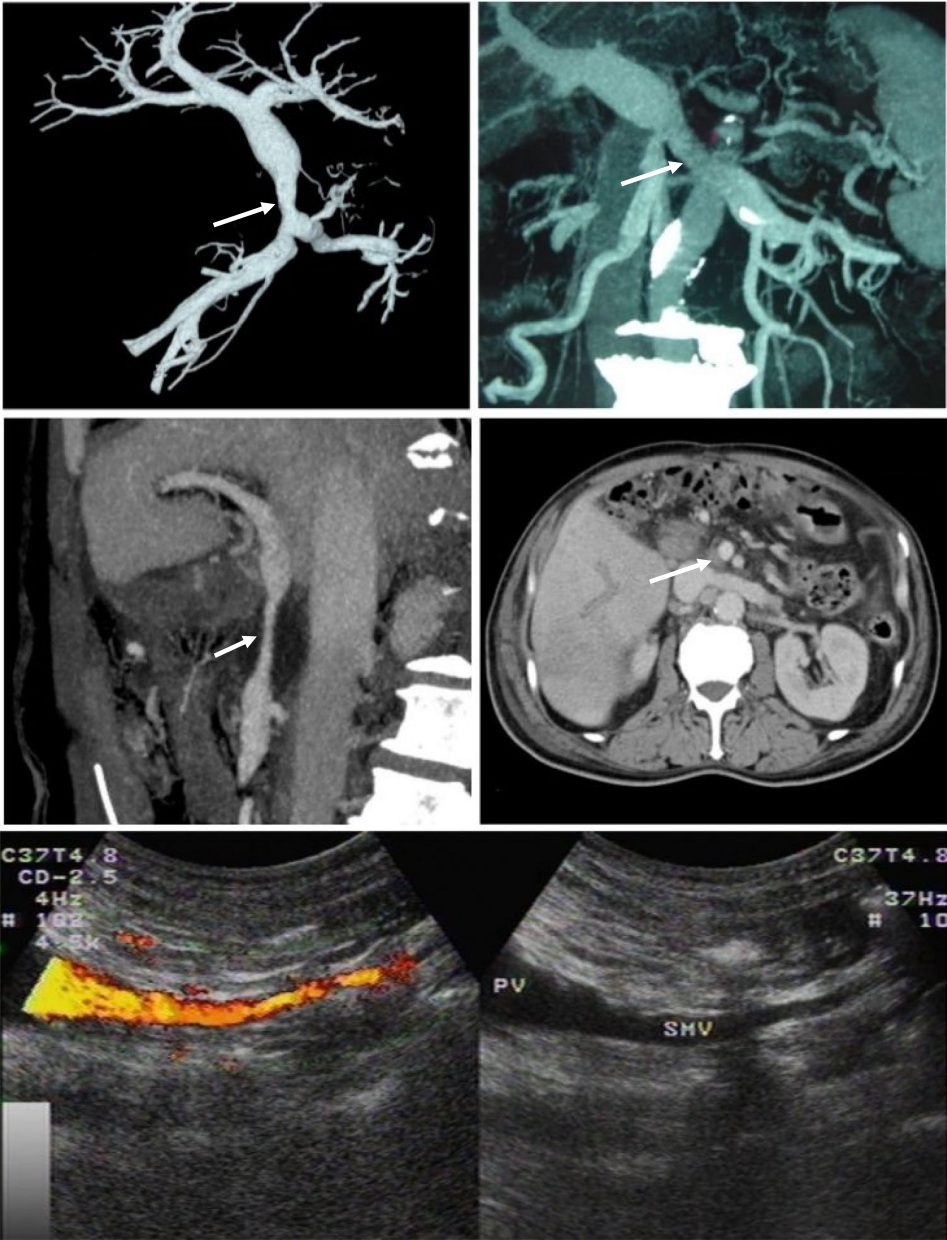
Images of the reconstructed portal vein and/or superior mesenteric vein. **a** Postoperative 1-month enhanced computed tomography (CT) reconstruction image of ligamentum teres hepatis (LTH) patch graft for portal-superior mesenteric vein (PSMV) reconstruction; **b** postoperative 3-month enhanced CT reconstruction image of LTH interposition graft for superior mesenteric vein (SMV) reconstruction; **c** postoperative 6-month coronal CT images of LTH patch graft for PSMV reconstruction; **d** postoperative 1-year sagittal CT images of LTH patch graft for PSMV reconstruction; **e** postoperative 2-week doppler ultrasound images of LTH graft for reconstruction

The three groups were followed to assess postoperative survival outcomes, excluding cases of 30-day postoperative mortality. Propensity score matching was performed using sex, age, and tumor stage as covariates for patients with pancreatic cancer in the PD-VRR group and the PD group, employing the nearest neighbor matching method at a 1:1 ratio. To ensure matching precision, we applied a clamping value of 0.03 for the age variable and performed exact matching for sex and tumor stage. Postoperative overall survival times were then compared between the matched cohorts. Color Doppler ultrasonography was performed after 2 weeks postoperatively to evaluate early vascular or graft patency and thrombus formation. Patients were followed up at 1, 3, and 6 months postoperatively, and every 6 months thereafter, until death. Enhanced computed tomography (CT) was performed during each follow-up to assess the reconstructed vessel (Fig. 3). The severity of stenosis in the reconstructed veins was evaluated using the classification system proposed by Kleive et al.^15^ The final follow-up was conducted on December 31, 2024.

### Statistical Analysis

All statistical analyses were performed using SPSS software (version 27.0; IBM Corp., Armonk, NY, USA). Data on patient age, operative time, intraoperative blood loss, and postoperative hospital stay were analyzed using the Mann–Whitney U test. Postoperative complication incidence was assessed using Pearson’s chi-squared test, and 30-day postoperative mortality was evaluated using the continuity-corrected chi-squared test. Overall survival curves were constructed using the Kaplan–Meier method. Descriptive statistics were summarized as mean ± standard deviation or median (range) for continuous variables and as percentages for categorical variables. Statistical significance was set at *p* < 0.05.

## Results

### Demographic and Clinical Characteristics of the Study Patients

The PD group comprised 336 patients (216 men, 120 women), with a mean age of 60.46 ± 9.75 (range 25–80) years. Diagnosis in this group included 103 cases of cholangiocarcinoma, 85 of pancreatic neoplasms, 92 of duodenal neoplasms, and 56 of ampullary neoplasms. The PD-VR group consisted of 23 patients (17 men, 6 women), with a mean age of 63.09 ± 9.33 (range 38–76) years, including nine cases of cholangiocarcinoma, 11 pancreatic tumors, and three duodenal tumors. The PD-VRR group comprised 28 patients (14 men, 14 women), with a mean age of 60.07 ± 9.49 (range 40–74) years, including four cases of distal cholangiocarcinoma, 20 pancreatic tumors, and four ampullary tumors. Statistical analysis revealed no significant differences in mean age among the groups (*p* = 0.407; Table 1).

**Table 1:** Basic characteristics of 387 patients

Characteristics	PD group (*n* = 336)	PD-VR group (*n* = 23)	PD-VRR group (*n* = 28)
Age, years	60.46 ± 9.75	63.09 ± 9.33	60.07 ± 9.49
Sex			
Male	216 (64.29)	17 (73.91)	14 (50.00)
Female	120 (35.71)	6 (26.09)	14 (50.00)
Tumor type			
*Cholangiocarcinoma*	103 (30.65)	9 (39.13)	4 (14.29)
Stage I	6 (1.79)	0	0
Stage II	24 (7.14)	1 (4.35)	0
Stage III	73 (21.73)	8 (34.78)	4 (14.29)
*Pancreatic tumor*	85 (25.30)	11 (47.83)	20 (71.43)
Stage I	20 (5.95)	0	0
Stage II	62 (18.45)	11 (47.83)	20 (71.43)
Stage III	3 (0.89)	0	0
*Duodenal tumor*	92 (27.38)	3 (13.04)	0 (0)
Stage I	19 (5.65)	0	0
Stage II	45 (13.39)	0	0
Stage III	28 (8.33)	3 (13.04)	0
*Ampullary tumor*	56 (16.67)	0	4 (14.29)
Stage I	3 (0.89)	0	0
Stage II	31 (9.23)	0	4 (14.29)
Stage Ⅲ	22 (6.55)	0	0

Data are presented as mean ± standard deviation or *n* (%)

PD, pancreaticoduodenectomy; PD-VR, PD with portal vein/superior mesenteric vein resection and reconstruction via end-to-end anastomoses or lateral venorrhaphy (PD with vascular resection group); PD-VRR, PD combined with portal vein/superior mesenteric vein resection and reconstruction using the autologous ligamentum teres hepatis graft (PD with vascular resection and reconstruction group)

### Intraoperative Data

The mean operative duration in the PD group was 371.02 ± 108.82 (range 170–715) min, and the mean intraoperative blood loss was 213.93 ± 195.09 (range 20–1600) mL. In the PD-VR group, 21 patients underwent PD combined with PV resection, and two underwent PD combined with SMV resection. Venous reconstruction was performed using end-to-end anastomosis in seven patients and lateral venorrhaphy in 16. The mean operative duration in the PD-VR group was 382.96 ± 113.53 (range 190–670) min, and the mean intraoperative blood loss was 223.91 ± 146.84 (range 50–600) mL. In the PD-VRR group, the LTH was used as an autologous graft to reconstruct the PV in 16 patients, SMV in 10, and portal-superior mesenteric vein in two. The graft was used as a lateral patch in six patients and an interposition graft in 22. The mean operative duration in the PD-VRR group was 484.86 ± 103.77 (range 285–685) min, mean portal or superior mesenteric vein clamp time of the PV/SMV was 51.79 ± 14.00 (range 25–85) min, and mean intraoperative blood loss was 236.79 ± 141.95 (range 80–800) mL. Operative duration was significantly longer in the PD-VRR group than in the PD and PD-VR groups; however, no significant differences in intraoperative blood loss were found among the groups (Table 2).

**Table 2:** Intraoperative and postoperative data of 387 patients

Variable	PD group (*n* = 336)	PD-VR group (*n* = 23)	PD-VRR group (*n* = 28)	*p*-value
Operation time, min	371.02 ± 108.82	382.96 ± 113.53	484.86 ± 103.77	<0.001
Intraoperative blood loss, mL	213.93 ± 195.09	223.91 ± 146.84	236.79 ± 141.95	0.116
Postoperative hospital stay, days	20.71 ± 9.78	18.30 ± 7.46	20.82 ± 8.25	0.366

Data are presented as mean ± standard deviation unless otherwise indicated

PD, pancreaticoduodenectomy; PD-VR, PD with portal vein/superior mesenteric vein resection and reconstruction via end-to-end anastomoses or lateral venorrhaphy (PD with vascular resection group); PD-VRR, PD combined with portal vein/superior mesenteric vein resection and reconstruction using the autologous ligamentum teres hepatis graft (PD with vascular resection and reconstruction group)

### Postoperative Complications, 30-Day Mortality Rate, and Postoperative Hospital Stay

In the PD group, the incidences of postoperative complications, 30-day mortality, and postoperative hospital stay were 31.85%, 5.06%, and 20.71 ± 9.78 (range 1–70) days, respectively. In the PD-VR group, the incidence of postoperative complications and the duration of postoperative hospital stay were 34.78% and 18.30 ± 7.46 (range 10–41) days, respectively. No perioperative mortality was recorded within the 30-day postoperative period. In the PD-VRR group, the incidence of postoperative complications was 42.86% (with no graft-related complications observed), the 30-day mortality rate was 7.14%, and postoperative hospital stay was 20.82 ± 8.25 (range 9–49) days. Statistical analysis revealed no significant differences in the incidences of postoperative complications, 30-day mortality rates, and postoperative hospital stay among the groups (Tables 2 and 3).

**Table 3:** Incidence of postoperative complications in the three groups

Postoperative complications	PD	PD-VR	PD-VRR
*Grade I*	20 (5.95)	5 (21.74)	5 (17.86)
Infectious complications	4	1	0
Bile leakage	11	2	3
Pancreatic leakage	2	1	0
Chylous leakage	3	1	2
*Grade II*	44 (13.1)	3 (13.04)	4 (14.29)
Infectious complications	6	0	1
Delayed gastric emptying	12	1	1
Bile leakage	19	1	0
Pancreatic leakage	2	0	0
Chylous leakage	4	0	0
Hemorrhagic complications	1	1	2
*Grade III*	18 (5.36)	0 (0.00)	1 (3.57)
Infectious complications	4	0	1
Hemorrhagic complications	3	0	0
Bile leakage	3	0	0
Pancreatic leakage	5	0	0
Small intestinal leakage	1	0	0
Delayed gastric emptying	2	0	0
*Grade IV*	11 (3.27)	0 (0.00)	0 (0.00)
*Grade V*: death	17 (5.06)	0 (0.00)	2 (7.14)
Incidence of postoperative complications (%)	31.85	34.78	42.86
Postoperative 30-day mortality (%)	5.06	0.00	7.14

Data are presented as *n* (%) or *n* unless otherwise indicated

PD, pancreaticoduodenectomy; PD-VR, PD with portal vein/superior mesenteric vein resection and reconstruction via end-to-end anastomoses or lateral venorrhaphy (PD with vascular resection group); PD-VRR, PD combined with portal vein/superior mesenteric vein resection and reconstruction using the autologous ligamentum teres hepatis graft (PD with vascular resection and reconstruction group)

### Postoperative Overall Survival and Follow-Up

**Fig. 4:**
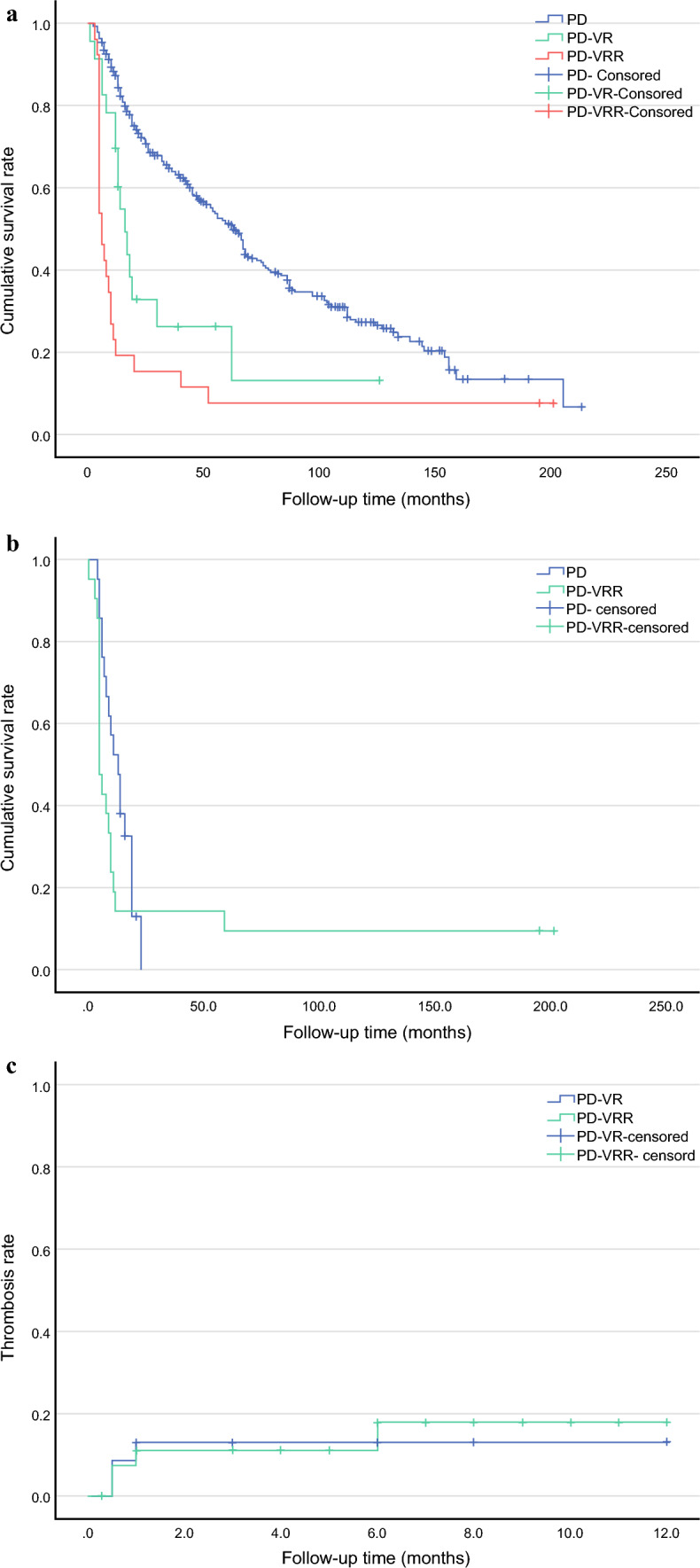
Postoperative follow-up results. **a** Overall survival of patients in the three groups; **b** overall survival of patients in the two subgroups; **c** thrombosis rates of portal vein/superior mesenteric vein reconstruction. PD-VR, pancreaticoduodenectomy with portal vein or superior mesenteric vein resection and reconstruction via end-to-end anastomosis or lateral venorrhaphy; PD-VRR, pancreaticoduodenectomy combined with portal vein or superior mesenteric vein resection and reconstruction using the autologous ligamentum teres hepatis graft

The median follow-up duration for the entire cohort was 106 (range 6–261) months, with 100 (range 6–261) months for the PD group, 82 (range 12–157) months for the PD-VR group, and 187 (range 25–255) months for the PD-VRR group. The median overall survival was 41 months (range 1 day–213 months) in the PD group, 13 (range 1–126) months in PD-VR group, and 6 (range 3–201) months in PD-VRR group. The overall survival duration in the PD group was significantly longer than that in the PD-VRR group (*p* < 0.05); no significant differences in survival outcomes were observed among other intergroup comparisons (all *p* > 0.05; Fig. 4a). The median overall survival of patients with pancreatic cancer in the PD-VRR group was 5 (range 3–201) months, and that of matched patients in the PD group was 13 (range 4–23) months, with no significant difference between the two groups (*p* > 0.05; Fig. 4b).

### Vascular Patency

In the PD-VR group, thrombosis within the reconstructed vascular grafts was detected in two patients using contrast-enhanced CT within 14 PODs. Two additional patients who underwent direct lateral suture repair developed postoperative vascular stenosis without associated thrombosis. All four cases of vascular stenosis were classified as grade A. In the PD-VRR group, early SMV thrombosis was identified in two patients at POD 14 following LTH interposition grafting. Additionally, delayed thrombotic complications were observed in two patients: one developed PV thrombosis at 3 months and the other developed SMV thrombosis at 6 months postoperatively, both events occurred in the context of LTH being used as an interposition graft. Postoperative imaging at 6 months demonstrated no evidence of thrombosis within the reconstructed vascular grafts. Among patients who developed thrombosis, the circumferential extent of the thrombus was limited to <50% of the vascular circumference and was classified as grade A stenosis (≤50% luminal narrowing). All cases resolved without venous occlusion following the initiation of therapeutic anticoagulation. The graft patency rate remained at 100%. The thrombosis rates (defined as having residual thrombus within the PV/SMV lumen but with patent graft) were 7.14%, 7.14%, 10.71%, and 14.29% at 2 weeks, 1 month, 3 months, and 6 months postoperatively, respectively (Fig. 4c).

## Discussion

This retrospective study analyzed 28 patients who underwent PD with venous resection using the LTH as a vascular graft. These patients were compared with 336 patients who underwent standard PD and 23 who underwent PD with venous resection followed by either direct end-to-end anastomoses or lateral venorrhaphy. The results indicated that, apart from the longer operative duration, there were no significant differences in postoperative complications, mortality rates, or hospital stay between the groups, suggesting that the procedure is safe and does not increase surgical risk. Two fatalities occurred within the PD-VRR group: one owing to abdominal hemorrhage resulting from intra-abdominal infection caused by a postoperative pancreatic fistula, and the other owing to abdominal hemorrhage (specific cause unknown). Postoperative analysis indicated that neither case was related to the graft material itself (i.e., neither was caused by material rupture, necrosis, rejection, or infection). Furthermore, this study provides long-term follow-up data for LTH grafts. Our findings demonstrate that they offer stable long-term patency rates and correlate with encouraging long-term survival rates. Notably, one patient achieved an overall survival of >13 years, providing compelling evidence for the long-term suitability of LTH as a reliable human vascular substitute.

Current options for vascular reconstruction grafts include several categories: artificial blood vessels,^3,16^ autologous vascular grafts (e.g., saphenous,^17–19^ gonadal/ovarian,^20–22^ internal jugular,^23^ femoral,^24^ iliac,^25^ and left renal veins^26,27^), allogeneic venous grafts,^6,28^ and alternative tissue materials such as falciform ligament and peritoneum^8,29,30^). However, these graft materials are associated with inherent limitations. The LTH retains the fundamental structural characteristics of a vein. Previous studies have demonstrated that the LTH provides optimal dimensional compatibility for PV/SMV reconstruction in terms of length and luminal diameter.^31^ Histologically, the tubular wall of the LTH contains elastic fibers, collagen fibers, and smooth muscle components similar to those found in the PV and SMV; additionally, the luminal endothelial cells express endothelial-type nitric oxide synthase and tissue-type fibrinogen activator.^12,32^ Our center has employed autologous LTH grafts for PV/SMV/IVC reconstruction since 2003, demonstrating favorable clinical efficacy.^10,11^ Recent literature has reported successful cases of major abdominal venous reconstruction using the LTH.^4,33–35^ Rochon et al.,^33^ performed mechanical recanalization of umbilical vein grafts for PV and SMV reconstruction in 14 patients; postoperative surveillance revealed no occurrences of graft-related infections or clinically significant hemorrhagic complications. Jiang et al.^36^ used dilated and recanalized LTH grafts for reconstruction of the right HV (RHV), left HV, middle HV, and IVC, resulting in favorable clinical outcomes.

Graft patency serves as a key parameter in assessing the efficacy of vascular grafts. Thrombosis remains a common complication following vascular reconstruction. Early thrombus formation, which causes significant luminal stenosis, may lead to graft failure. Liao et al.^37^ used polytetrafluoroethylene prosthetic vessels for SMV-PV reconstruction in 34 patients, achieving a postoperative patency rate of 83.5% at the 12 months. In studies using autologous veins for PV/SMV,^18,38^ postoperative patency rates were 61–88%. Reported thrombosis rates are even higher in reconstructions utilizing allogeneic vein grafts. Kleive et al.^15^ used cryopreserved allograft vessels for PV and SMV reconstruction, with postoperative thrombosis observed in both grafts. Rochon et al.^33^ demonstrated that the use of LTH for PV, SMV, and HV reconstruction yielded an 80% vessel patency rate. Ikegami et al.^39^ found that the use of dilatation-recanalized LTH as a vein patch graft for RHV reconstruction preserved endothelial cell integrity and hemodynamic function, maintaining luminal patency without postprocedural thrombosis or graft-related stenosis. In the present study, PV and SMV reconstruction using LTH resulted in a vascular patency rate of 100%. Although four patients developed intragraft thrombosis postoperatively, the resultant vessel stenosis was classified as grade A. Severe symptoms of portal hypertension, including intestinal stasis, and hepatic dysfunction were absent, and thrombi resolved following anticoagulation therapy. No new instances of graft thrombosis were observed during the 6-month postoperative period. These findings suggest that high vascular patency rates can be achieved following PV/SMV reconstruction using LTH as an autologous graft under standardized postoperative anticoagulation management. In this study, postoperative imaging revealed relatively narrow lumens in some LTH grafts, possibly related to the microscopic structural characteristics of the graft walls. LTH comprises elastic and collagen fibers, whose arrangement and subtle inter-individual variations in fiber content may influence morphology under prolonged hemodynamic stress, leading to luminal differences between patients. However, this radiographic relative stenosis did not translate into clinical adverse outcomes. We observed no typical signs of portal hypertension-induced splenomegaly, hypersplenism, ascites, or collateral circulation formation. This indicates that, within the current observation period, the lumen diameter of LTH grafts is sufficient to maintain adequate hepatic blood flow, meeting the functional demands of the liver. Furthermore, another noteworthy finding was that no graft thrombosis was observed in patients (*n* = 6) undergoing patch reconstruction, with superior patency compared with the segmental reconstruction group. We hypothesize that this advantage may stem from the inherent characteristics of the patch technique. First, from a hemodynamic perspective, the patch approach involves only local modification of the vessel wall, maximally preserving the native vessel's anatomical structure and physiological course. This may consequently generate a blood flow pattern more conducive to laminar flow, thereby reducing prothrombotic factors such as turbulence and eddies. Second, this technique requires only unilateral incision and suturing of the vessel wall; it causes less extensive trauma to the vascular endothelium and milder endothelial dysfunction than the circular end-to-end anastomosis necessitated by segmental resection. This facilitates faster restoration of the anticoagulant and antiplatelet aggregation functions. Therefore, prioritizing patch reconstruction techniques that cause less disruption to vascular continuity may represent a favorable strategy for reducing postoperative thrombotic risk.

In this study, the overall survival duration of patients in the PD-VRR group was shorter than that of those in the PD group, attributed to a higher proportion of pancreatic cancer cases and more advanced tumor stages among patients undergoing PD-VRR. To adjust for these confounding factors, a propensity score-matching analysis was performed. A subgroup analysis of patients with pancreatic cancer after matching showed no significant difference in survival between the PD-VRR and PD groups. These findings suggest that PD combined with PV or SMV resection and reconstruction may offer survival benefits to patients with pancreatic cancer and confirmed vascular invasion.

This study has certain limitations. We excluded cases requiring concomitant resection of multiple organs, thereby implying that the generalizability of our findings to the most complex, locally advanced cases requiring combined multi-organ resection may be limited. Nevertheless, this choice was made as per the study's primary objective: to systematically evaluate the feasibility and safety of this novel graft under conditions wherein variables were relatively controllable. We believe this provides crucial foundational evidence for subsequent application of the technique in broader, more complex patient populations. Moreover, the sample size in this study was relatively limited. Although we employed Cox proportional hazards analysis and propensity score matching, the small sample size constrained the number of covariates we could include in the matching process. Furthermore, the limited sample size may have restricted the statistical power of certain subgroup analyses. Future multicenter, large-scale studies are needed to validate our findings.

Recanalized LTH grafts were used for PV and SMV reconstruction, demonstrating surgical safety and technical feasibility. The reconstructed vessels demonstrated a high patency rate. The advantages of using recanalized LTH for venous reconstruction include: (i) it is an autologous graft, thereby eliminating the risk of immune rejection; (ii) it can be harvested easily without additional incisions; (iii) it is safe, with no complications post-resection and no secondary injury; (iv) its luminal diameter closely matches that of the portal or superior mesenteric vein; and (v) it is resource-efficient, involving no additional cost.

In patients undergoing open abdominal surgery, the LTH is often excised without specific indication, necessitating the use of autologous veins or alternative graft materials for revascularization, which consequently increases patient morbidity and healthcare costs. Therefore, hepatobiliary surgeons should prioritize preserving the LTH for potential use for vascular reconstruction in emergency situations. Future studies should prioritize prospective comparative research and include larger sample sizes to provide more robust clinical evidence supporting the use of LTH as an autologous graft for PV/SMV reconstruction.

## Conclusion

The use of artificially recanalized LTH as a vascular substitute for PV/SMV reconstruction during PD with PV/SMV resection was safe and technically feasible. Under standardized postoperative anticoagulation therapy, the grafted vessels maintained a high postoperative patency rate.

## Supplementary Information

Below is the link to the electronic supplementary material.

Supplementary file1 (MP4 49423 KB)

Supplementary file2 (MP4 53517 KB)
